# Global meta‐analysis reveals the drivers of gut microbiome variation across vertebrates

**DOI:** 10.1002/imo2.35

**Published:** 2024-10-04

**Authors:** Yong Xie, Songsong Xu, Yufei Xi, Zixin Li, Erwei Zuo, Kai Xing, Lijing Bai, Kui Li

**Affiliations:** ^1^ Shenzhen Branch, Guangdong Laboratory of Lingnan Modern Agriculture, Agricultural Genomics Institute at Shenzhen Chinese Academy of Agricultural Sciences Shenzhen China; ^2^ College of Animal Science and Technology China Agricultural University Beijing China; ^3^ Animal Science and Technology College Beijing University of Agriculture Beijing China; ^4^ Animal Breeding and Genomics Wageningen University & Research Wageningen The Netherlands

**Keywords:** antibiotic resistomes, climate variation, diet, gut microbiome, microbial diversity

## Abstract

Shifts in gut microbial diversity and structure are one route by which vertebrate hosts adapt to local environmental conditions. However, recent studies have mostly been limited to a single species, small sample sizes, or restricted geographic ranges. Therefore, drawing a global picture of vertebrate gut microbiome diversity, community structure, and determinants for their adaptive shifts remains to be elucidated. We here collected 6508 samples from 113 vertebrate species covering diverse classes, feeding behaviors, and host habitats based on 16S rRNA gene sequencing. The results showed that host diet pattern had a significant impact on gut microbiome variation, which might drive taxonomic and functional contents of gut microbiome across vertebrates. Of note, the phylum Fusobacteria were enriched in carnivorous vertebrate gut while herbivorous vertebrate gut selectively increased the abundance of Verrucomicrobia. Also, climate factors were strongly associated with gut microbiome variation across vertebrates. Interestingly, we found that the abundance of microbiota belonging to Bacteroidetes increased gradually while the members from Proteobacteria showed a decreasing trend from high‐ to low‐latitude zones, potentially contributing to vertebrate adaptation to local climate condition. Additionally, we comprehensively deciphered the common antibiotic resistomes and their potential mobility between terrestrial vertebrate gut microbiome (*n* = 487) and their sympatric soil biological environment samples (*n* = 203) by integrating metagenomic sequencing datasets. Particularly, potential horizontal antibiotic resistance genes (e.g., *bacA*) transfers were detected between vertebrates gut microbiome and their sympatric soil biological environment. Together, our findings provide new evidence of how external environmental factors affect vertebrate gut microbiome variation.

## Highlights

- Host diet and habitat climate factors can strongly influence the composition and function of the gut microbiome.
- Vertebrate gut microbial diversity increases with decreasing habitat latitude.
- The abundance of Bacteroidetes and Proteobacteria in the gut microbiome was greatly influenced by climate factors.
- Horizontal transfer of potential resistance genes exists between vertebrates and sympatric soil.

## Graphical abstract

This study analyzed 6508 16S rRNA gene sequence data from 113 vertebrate species worldwide and evaluated the effects of host diet and habitat climate on the diversity of the vertebrate gut microbiome. The results showed that diet and climate factors significantly affected changes in the gut microbiome. At the same time, we also investigated 690 metagenomic samples and found horizontal transfer of antibiotic resistance genes (such as *bacA*) between the gut microbiome of vertebrates and the sympatric soil environment. This study provides new insights into the diversity and resistance characteristics of the vertebrate gut microbiota and is of great significance for animal protection and antibiotic management.

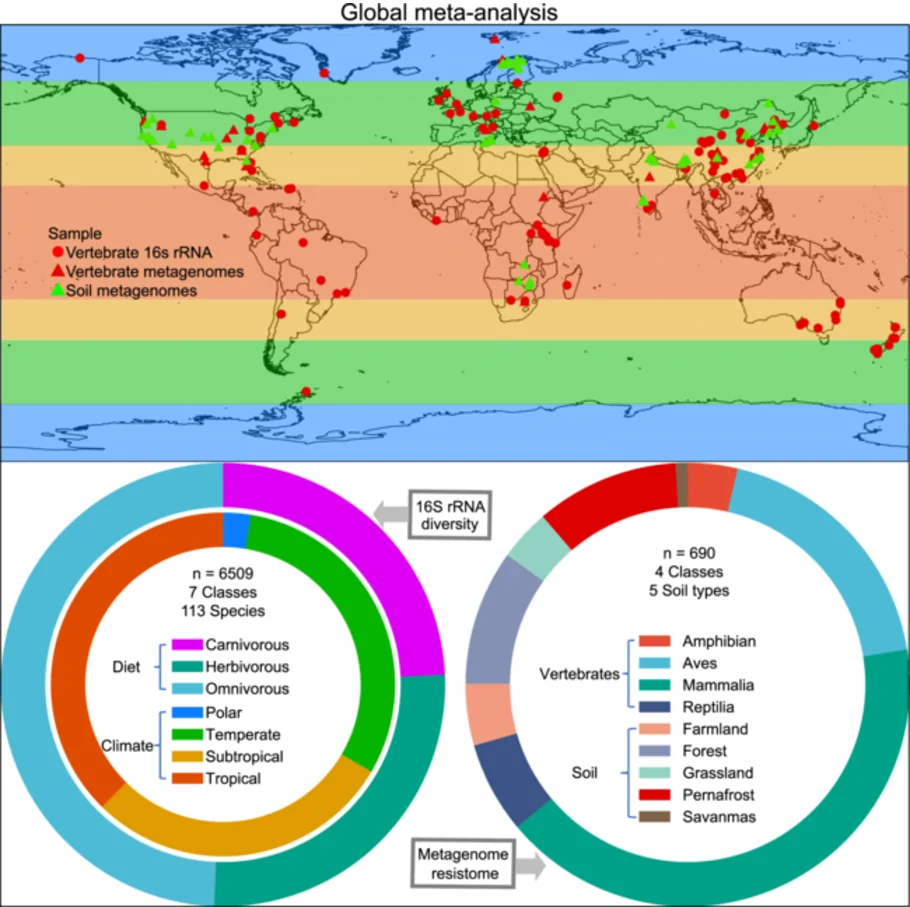

## 1 INTRODUCTION

Adaptation is one of the most striking features of biological organisms and an essential capability for survival in diverse environments [1]. Together with other sources, such as host standing variation and mutations, the trillions of microorganisms inhabiting animal guts may drive adaptive benefits [2]. The gut microbiota could facilitate the diversification of host dietary niches, altering future host adaptive trajectories [3]. For instance, the gut microbiota of koalas, specialized in eating Eucalyptus, harbor bacterial functional pathways associated with the digestion of plant secondary metabolites in a higher measure than the microbiome of wombats, a sister species [4, 5].

Shifts in the gut microbiota may also affect host physiological and metabolic function, such as body mass index [6], visceral fat [7], and metabolic syndrome [8] in humans, as well as feed efficiency [9], methane emissions, rumen, and blood metabolites, and milk production efficiency in cattle [10]. It has been hypothesized the existence of selective pressures that could result in the reshaping of the microbiota to benefit host fitness [3]. For example, Toll‐like receptor deficiency in mice exacerbates intestinal dysbacteriosis, which negatively contributes to the metabolism and ability of host to harvest energy [11]. Yet a comprehensive understanding of the contribution of gut microbiome to adaptive evolution in vertebrates has not been achieved, as most recent studies have been limited to one species, a small number of samples, or confined geographic distributions.

The composition of the gut microbiota is influenced by a variety of intrinsic and extrinsic factors, among which the host's dietary pattern, phylogeny [12, 13, 14, 15], social interactions [16, 17], and environmental factors such as climate and soil are particularly critical [18]. Climate change not only affects the physiological and immune functions of the host, but also affects its selection of microorganisms from the surrounding environment [19, 20]. In recent years, several studies have focused on the diet and phylogeny of hosts, which are known factors leading to changes in animal intestinal microbiota [21, 22, 23], while environmental factors and responses to different local climates have rarely been investigated. Although there are several reports regarding the effect of climate variation on gut microbial communities [24, 25, 26], few studies have reported on the relationship between climate factors and vertebrate gut microbiota on the global landscape. Soil, as an important external factor that the host may be exposed to, also provides a source of diversity for the microbiota [27]. At the same time, other factors such as antibiotic exposure and gastrointestinal dysfunction can also lead to changes in the gut microbiota of vertebrates [28, 29]. In addition, studies have shown that mobile genetic elements (such as plasmids, transposons, etc.) carrying resistance genes can move between soil microorganisms and the intestinal microbiota of vertebrates [30, 31, 32], especially in situations where the host frequently contacts soil, which may exacerbate the problem of antibiotic resistance worldwide. For example, Chen et al. used soil collembolan (*Folsomia candida*)‐predatory mite (*Hypoaspis aculeifer*) as the model food chain, and observed that the application of manure significantly increased the drug‐resistant genes in the microbial communities of springtails and predatory mites, caused resistance gene transfer, changed the microbial community structure, and reduced microbial diversity [33]. By combining these two external factors, climate and soil environment, we can not only better understand their impacts on the gut microbiota, but also reveal how these factors further affect the health and fitness of the host by affecting the composition and function of the microbiota.

In the present study, we collected 6508 samples of 16S rRNA gene sequence from all seven classes of vertebrates (*Mammalia*, *Aves*, *Reptiles*, *Amphibia*, *Actinopterygii*, *Chondrichthyes*, and *Cyclostomata*). We further collected data for metagenomic sequences, including 487 terrestrial vertebrate gut microbiomes and 203 sympatric soil biological environment samples. The aims of the study are: (1) assess the effects of environmental factors on vertebrate gut microbiome diversity; (2) characterize the patterns of vertebrate gut microbiome composition and function, both in terms of host diet pattern and habitats; (3) identify potential horizontal transfer of antibiotics resistance genes (ARGs) between vertebrate gut microbiomes and sympatric soil samples.

## 2 RESULTS

### 2.1 Overview of data related to the vertebrate gut microbiome

To comprehensively depict the gut microbiomes of vertebrates, we analyzed the diversity and composition of the gut microbial communities in 113 species of vertebrates, including *Mammals* (*n* = 4668 from 60 species), *Aves* (*n* = 792 from 25 species), *Reptiles* (*n* = 347 from 10 species), *Amphibia* (*n* = 104 from five species), *Actinopterygii* (*n* = 520 from nine species), *Chondrichthyes* (*n* = 33 from two species), and *Cyclostomata* (*n* = 44 from three species) (Figure S1). The samples were collected from vertebrates inhabiting seven continents and with different diets, habitat types, captive status, threatened status and climate zones (Table S1).

**Figure 1:**
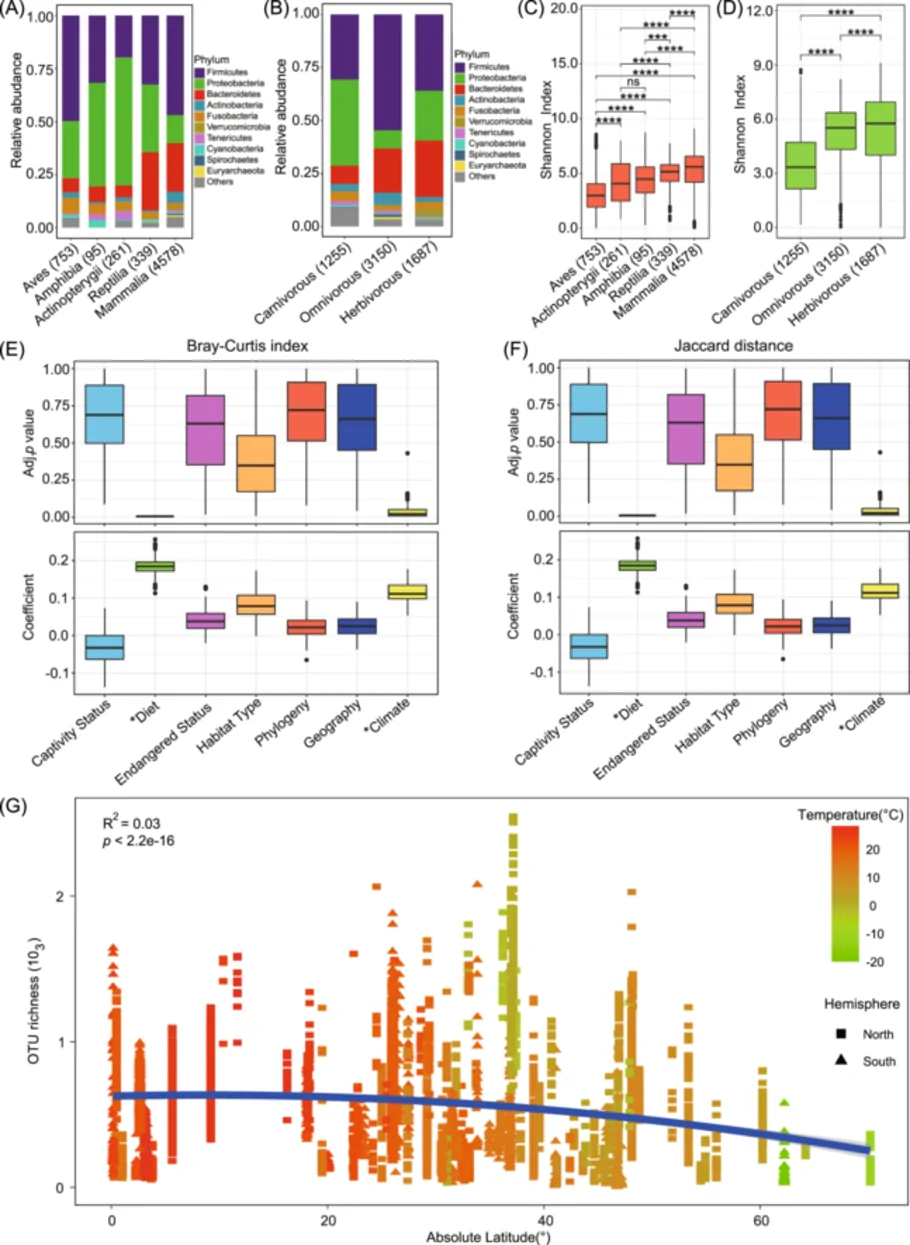
Evaluation of the effects of different factors on the diversity of vertebrate gut microbial communities. (A and B) Stacked bar plot of microbiome distribution at the phylum taxonomic level showing the top 10 categories within each grouping, with the remaining categories shown in gray, grouped by host vertebrate class (A) and diet (B). (C and D) Alpha diversity levels in gut communities grouped by host vertebrate class (C) and diet (D). False discovery rate (FDR)‐corrected Wilcoxon rank sum tests were used to determine significance. ***: *p* < 0.001, **: *p* < 0.01, *: *p* < 0.05. (E and F) The plots show the BH‐adjusted *p*‐values (Adj. *p* value) and partial regression coefficients (Coef.) for multiple regression on matrix (MRM) tests to determine the extent to which host diet, captivity status, geographic location, habitat type, phylogeny, climate, and threat status explain the variance in Bray‐Curtis index (E) and Jaccard distance (F). The boxplots show the distributions of coefficients and adjusted *p* values obtained from the Multiple regressions on matrices (MRMs) tests for each of 100 random data subsets (each subsample including only one sample per species). Asterisk denotes significance (Adj. *p* < 0.05 for ≥95% of data set subsets). Box centerlines, edges, whiskers, and points signify the median, interquartile range (IQR), 1.5 × IQR, and > 1.5 × IQR, respectively. (G) Latitudinal distribution of vertebrate gut diversity. The best polynomial fit was determined on the basis of the corrected Akaike information criterion (AIC) for the given datasets in this study. The line shows the second‐order polynomial fit based on ordinary least‐squares regression (R^2^ = 0.03, *p* < 2.2e‐16). The color gradient represents the temperature at the collection location corresponding to each sample. Shapes of symbols denote whether a sample originated from the Northern Hemisphere (square) or the Southern Hemisphere (triangle). OTU, Operational taxonomic units.

Overall, we obtained 39,613 operational taxonomic units (OTUs) from 1290 genera within 47 phyla. The phylum‐level relative abundances were compared among vertebrate lineages, revealing that *Proteobacteria* dominate the gut microbiota of aquatic and amphibian species (Figures 1A, S2). We also found that *Verrucomicrobia* were prevalent in the guts of herbivores, such as *Equus caballus*, *Phascolarctos cinereus*, *Ochotona curzoniae*, and *LCervus elaphus* (Figures 1B, S2).

The gut microbiome of different animal taxa may be shaped by environmental (i.e., habitat type, geography, and climate) and genetic factors. The extent to which these factors may contribute to the diversity and composition of vertebrate gut microbiomes is yet not fully understood. We first calculated the alpha diversity (i.e., a measure of microbiome diversity) of gut microbial communities using the Shannon index. The gut microbiota of mammals showed the highest level of alpha diversity (i.e., Shannon index = 5.26), while the gut microbiota of birds scored the lowest (i.e., Shannon index = 3.14) (Figure 1C). Furthermore, herbivores had the highest gut microbial diversity, while carnivores had the lowest (Figure 1D). In addition, wild vertebrates had higher diversity than captive populations, and the microbiomes of terrestrial vertebrates were more diverse than those of aquatic and amphibian species (Figure S3A). We also observed lower alpha diversity levels in threatened species (i.e., *Ailuropoda melanoleuca*) compared to non‐threatened species (i.e., *Ochotona curzoniae*) (Figure S3A). Finally, we compared the diversity of samples of different body sizes and gut lengths, with species with larger bodies and longer guts having higher microbial diversity (Figure S3A, Table S2).

We then performed Multiple regressions on matrices (MRMs) analysis to evaluate the importance of various factors affecting gut microbiome of vertebrates. Each of four MRM models (one per diversity metric) had a significant overall fit. Host diet and habitat climate were main significant explanatory variables (BH‐corrected *p* < 0.05, Figures 1E,F, S3B). Host diet explained a substantial amount of alpha‐ and beta‐ diversity variation (~8%–24%) and was significant for all diversity metrics tested (i.e., Shannon index, Bray‐Curtis, Observed Feature, and Jaccard distance). Habitat climate also explained a substantial amount of the variaḍtion in alpha‐ and beta‐ diversity (~11%–16%). In addition, we used random forests to determine the relative importance of different factors (Figure S4). Host diet and habitat climate factors were most important for the Alpha diversity of vertebrate gut microbes, while host geographic location was most important for the Beta diversity of vertebrate gut microbes. These results further demonstrated that gut microbiome variations in vertebrates are primarily driven by diet and climate factors.

To show the significant impact of host diet and habitat climate factors on microbial diversity, we first examined the intestinal microbial diversity of vertebrates of the same class or order with different dietary habits, and obtained the same results as Figure 1D (Figure S5A). Following this, we noticed that the greatest microbial diversity was observed at intermediate latitudes (Figure 1G, R^2^ = 0.03, *p* < 2e‐16). This was consistent with the two other major types of ecosystems on Earth, that is, ocean [34] and air [35]. At the same time, we can also notice that the microbial Shannon index increases year by year with the increase of sample collection years (Figure S5B, Table S3), while the global surface temperature increases year by year [36]. Therefore, temperature can be considered an important factor driving microbial diversity in the gut of vertebrates.

### 2.2 Changes in vertebrate gut microbial composition and function in response to diet and climate factors

**Figure 2:**
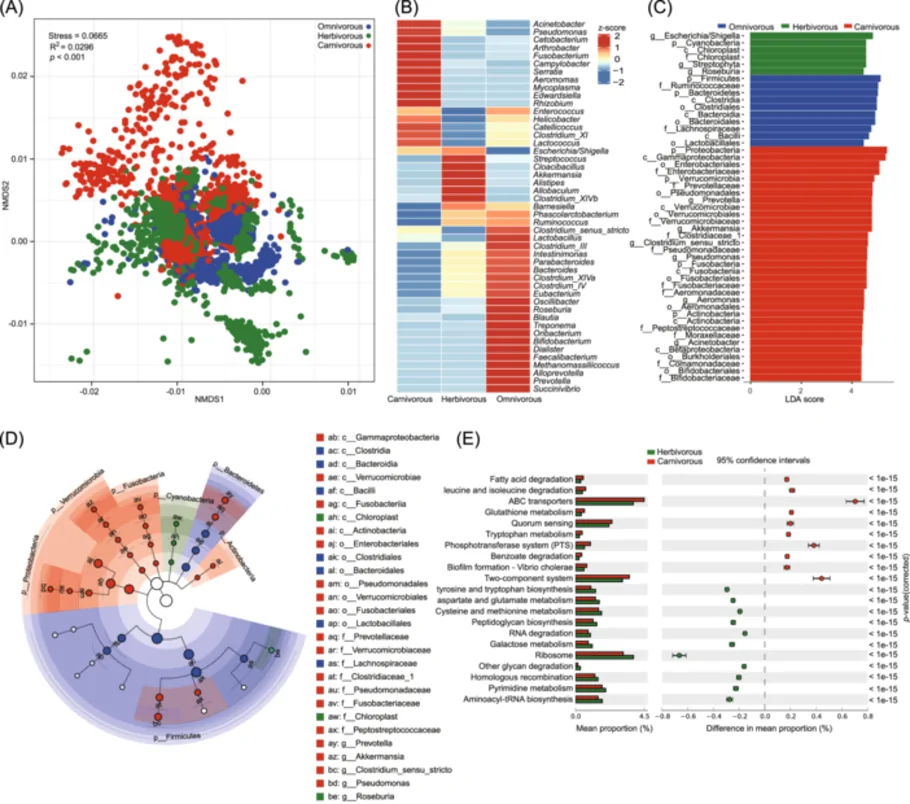
Impact of diet on the composition of vertebrate gut microbiota. (A) Bray–Curtis‐based nonmetric multidimensional scaling (NMDS) plot, which shows that each diet is associated with different gut bacterial communities in vertebrates. The Bray–Curtis distance was calculated to represent dissimilarities in the composition of bacterial communities. (B) Impact of diet on genus‐level taxonomic composition: distributions 47 genera with relative abundances >0.1% and present in ≥50% samples within gut communities of vertebrates with different diets. Genera highlighted in red have higher abundance, and those highlighted in blue have lower abundance. (C and D) Results of LEfSe analysis. (C) The histogram displays species whose Linear Discriminant Analysis (LDA) Score exceeds the default value (4). The length of the histogram bars represents the impact of the different diets. (D) Species that displayed no significant differences are marked in yellow. The colors represented by vertebrates with different diets are the same as those of the different dietary states in the evolutionary cladogram. (E) PICURSt2 functions prediction of microbiota in carnivores and herbivores. Functional differences between the two groups.

To evaluate the impact of dietary patterns on the composition and function of the vertebrate gut microbiome, we performed a nonmetric multidimensional scaling (NMDS) analysis based on the Bray‐Curtis distance. In the NMDS analysis, the most significant differences were found between carnivores and herbivores (Figure 2A, Table S4). We selected the gut microbiota with relative abundance higher than 0.1% in at least 50% of the individuals at genus level to generate a heatmap of species relative abundance (Figure 2B). We observed significant changes in microbial community composition corresponding to differences in host diet (Figure 2C,D). For example, the genera, including *Acinetobacter*, *Aeromonas*, and *Pseudomonas*, were enriched in carnivores, whereas the relative abundance of *Streptococcus* is higher in herbivores. The major KEGG (Kyoto Encyclopedia of Genes and Genomes) functional categories (level 3) found in the gut microbiomes of herbivores and carnivores include amino acid metabolism and synthesis, protein transport, and sugar metabolism, among others (Figure 2E). Notably, the gut microbiota of carnivores plays key roles in material transport, environmental adaptation, and antioxidant protection (e.g., ABC transporters, Two‐component system, Phosphotransferase system [PTS], and Glutathione metabolism), whereas the gut microbiota of herbivores is enriched in protein synthesis‐related pathways (e.g., ribosomes, aminoacyl‐IRNA biosynthesis, tyrosine and tryptophan biosynthesis) (Figure 2E). Our findings therefore confirm that diet is a critical factor shaping the composition and function of vertebrate gut microbiomes.

**Figure 3:**
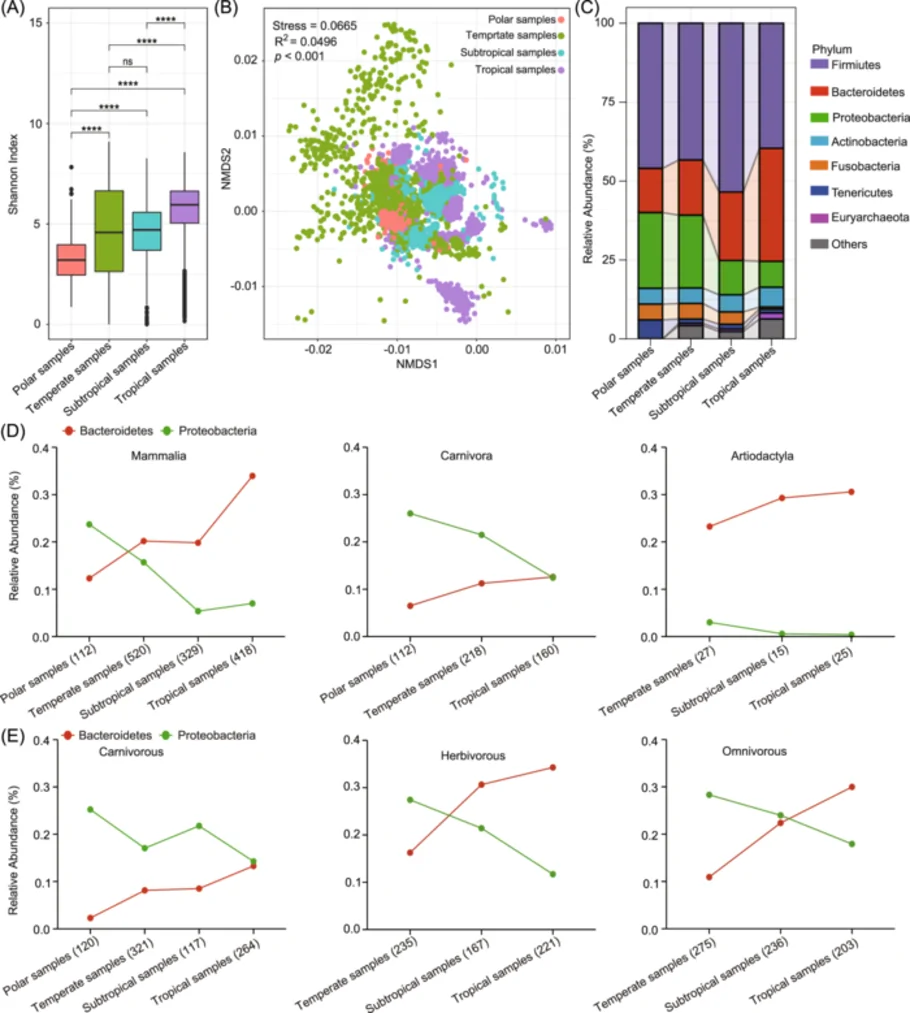
Effects of different climate regions on vertebrate gut microbial communities. (A and B) Alpha‐ (A) and beta‐ diversity (B) levels of gut microbial communities in vertebrates from different climatic regions. (C) Stacked bar plot of microbiome distribution at the phylum taxonomic level showing the top seven categories within each grouping, with the remaining categories shown in gray, grouped by the climate zone in which the hosts are located. (D) The relative abundance trends of Proteobacteria and Bacteroidetes in the intestinal tracts of vertebrates across different climate zones within the same class or order. (E) The relative abundance trends of Proteobacteria and Bacteroidetes in the intestinal tracts of vertebrates across different climate zones within the same diet.

Climate factors are also strong factors in the determination of gut microbiomes. Importantly, our analysis revealed that the microbiome diversity (i.e., Shannon index) increased gradually from high‐ to low‐latitude zones (Figures 3A, S6). We also detected differences in beta diversity (i.e., a measure of the similarity or dissimilarity of two communities) within the four climate regional samples. As shown in the NMDS plot, the polar samples clustered closely together, whereas temperate and tropical samples clustered less closely on NMDS1 (Figure 3B, Table S4), indicating more diverse gut microbial compositions in the low‐latitude region. Seven major microbial phyla dominated across the four climate zones: Firmicutes, Bacteroidetes, Proteobacteria, Actinobacteria, Fusobacteria, Tenericutes, and Euryarchaeota (Figure 3C), which together constituted up to 96.4% OTUs. Notably, the richness of Bacteroidetes phylum (class Bacteroidia, order Bacteroidales, family Prevotellaceae, and genus *Prevotella*) in gut microbiota of vertebrates increased from high‐ to low‐latitudes where the hosts reside (Figures 3C, S7). In contrast, the abundance of members of Proteobacteria phylum (class Gemmaproteobacteria and genus *Photobacterium*) decreased from high‐ to low‐latitude zones (Figures 3C, S7). In addition, we controlled for batch and individual study effects in the sequencing data to further illustrate the changing trends of Bacteroidetes and Proteobacteria in vertebrate gut microbiota in different climate regions (Figure 3D,E).

We also constructed co‐occurrence microbial networks to identify robust microbial association patterns in the gut of vertebrates across four climatic regions. Co‐occurrence network diagrams and related parameters, including the number of nodes and links, average clustering coefficient (avgCC), average path distance (GD), Average Degree (avgK), and modularity (M), for gut microbial communities in the four climatic regions were provided (Figure S8, Table S5). The results indicated that the complexity and stability of gut microbial community networks were highest to lowest in tropical, subtropical, temperate, and polar regions. Notably, the tropical co‐occurrence network contained the highest number of significantly co‐occurring OTUs, while the polar network contained the fewest. Compared to low‐latitude networks, the structure characteristics, nodes, and edges of co‐occurrence networks in high‐latitude regions were lower than those in other regions, suggesting that gut microbial communities in high‐latitude regions are more susceptible to climate disturbances. Additionally, from high to low latitudes, the co‐occurrence clusters with high betweenness centrality of Bacteroidetes gradually increased, whereas those of Proteobacteria gradually decreased, possibly reflecting adaptive changes to climatic variations. These results further confirm the known associations between climate factors and vertebrate gut microbiomes.

### 2.3 Gene mobility potentials of common antibiotic resistomes in the gut microbiome of vertebrates and their sympatric soil biological environment

**Figure 4:**
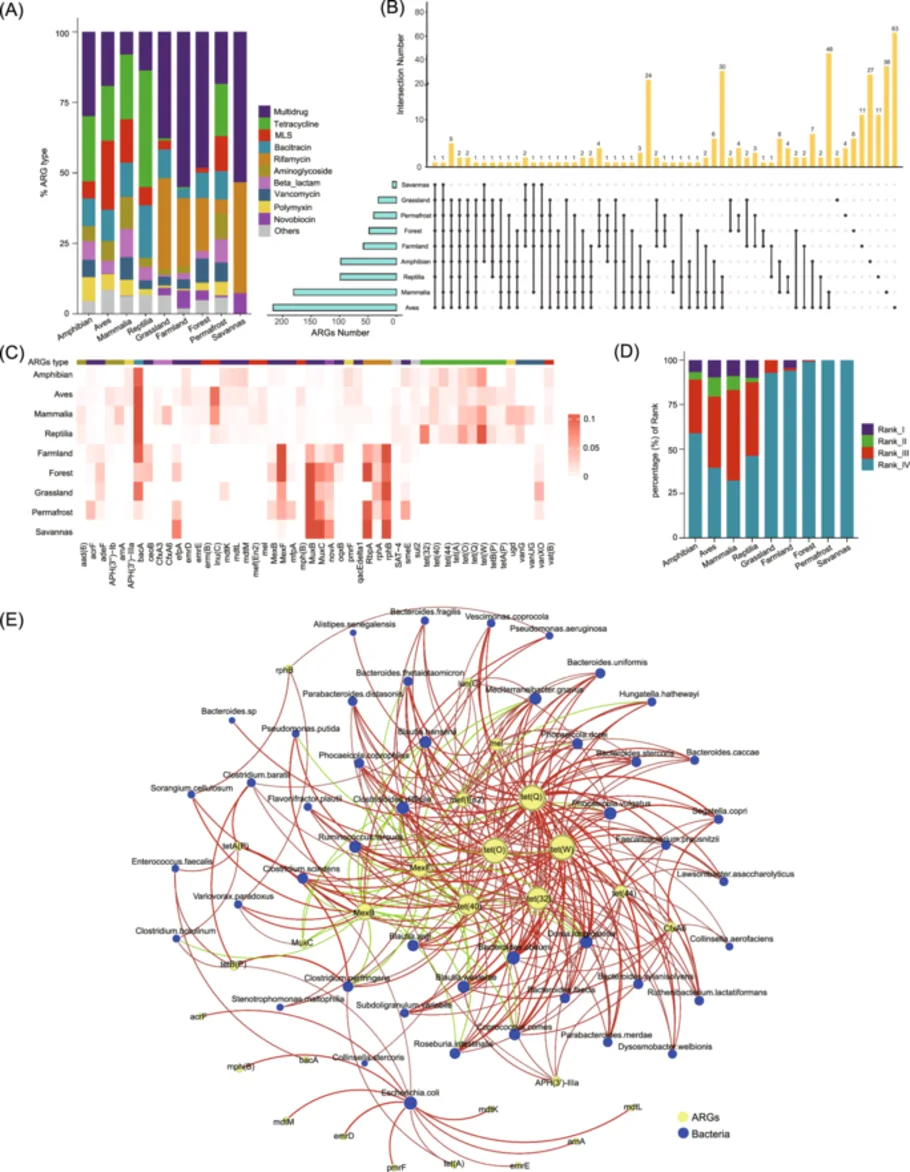
Antibiotic resistance genes in the gut microbiota of vertebrates and in soil. (A) Stacked bar plots showing the relative abundance of the top 10 antibiotic resistance gene types detected in each grouping, with the remaining types shown in gray. (B) Overlap of antibiotic resistance genes (ARGs) in vertebrate gut microbes and soil microbes. (C) Heat map showing the relative abundance of ARG subtypes covering 10 different resistance types in four vertebrate categories and five soil categories. The top 50 relative abundance ARG subtypes are shown. Red colors indicate the relative abundances of ARG subtypes based on the scale in the lower left. (D) The risk assessments of ARGs; the percentage (%) of Rank Ⅰ, Rank Ⅱ, Rank Ⅲ, and Rank Ⅳ are shown. MLS, Macrolide lincosamide streptogramin. (E) Network analysis revealed co‐occurrence patterns between ARG subtypes and microbial species in vertebrate gut samples and environmental samples. The nodes were colored according to ARG types and specie. A connection represents a strong (Spearman's correlation coefficient *ρ* > 0.6) and significant (*p*‐value < 0.05) correlation. The size of each node is proportional to the number of connections, that is, degree.

Soil biological samples have a large gene pool of antibiotic resistant bacteria. ARGs threaten vertebrates’ health worldwide, but the common resistome and ARGs mobility between vertebrate and their sympatric soil biological environment remain unclear. To fully decipher the common resistome and potential mobility of ARGs, we collected and analyzed 487 vertebrate gut microbial samples and 203 sympatric soil environment samples using metagenomic sequencing (Figure S1, Table S6). We found 80.8% ARGs belonged to the top types (i.e., multidrug, tetracycline, macrolide‐lincosamide‐streptogramin [MLS], bacitracin, rifamycin, aminoglycoside, beta_lactam, vancomycin, polymyxin, and novobiocin) Moreover, these types accounted for 89.1% of the total abundance of ARGs (Figure 4A). The 43 subtypes of ARGs, mainly comprising multidrug (*MexF*), tetracycline (*tet(S)*), MLS (*lmrD*), bacitracin (*bacA*), rifamycin (*RbpA*), aminoglycoside (*AAC(6’)‐Ii*), beta_lactam (*TEM‐116*), vancomycin (*vanI*), polymyxin (*arnA*), and novobiocin (*novA*) resistance genes, were shared between the vertebrate gut microbiomes and their sympatric soil biological environmental samples (Figure 4B). More specifically, a large number of overlapping ARGs (6.5%) were shared among aves and soil biological environmental samples (Figure 4B). We also observed the *bacA* (also known as *UppP*, undecaprenyl‐diphosphate or ‐pyrophosphate phosphatase) gene, which confers resistance to bacitracin, was abundant in both vertebrate and soil organism environmental samples (Figure 4C). In addition, to understand the threats posed by ARGs, an ARG‐ranker was used to classify the identified ARGs into four levels, and a total of 427 ARG subtypes were identified (Tables S7, S8). In the gut microbiota of birds, 20.6% of the ARGs detected were at risk level I‐II. In the gut microbiota of mammals, reptiles, and amphibians, the proportions were 16.8%, 12.4%, and 11%, respectively. In contrast, only 4.3% and 0.3% of ARGs at risk level I‐II were detected in farmland and forest soil types (Figure 4D). This observation suggests that most ARGs present in soil types are not enriched in human‐related environments (level Ⅳ), indicating a lower clinical risk.

The co‐occurrence network correlation between ARGs and bacterial groups is considered an effective method to track potential ARG hosts in various environments [37]. To explore the association between ARGs and bacterial communities, the correlation analysis of the relative abundance of ARGs and bacteria was performed. If there is a significant correlation between the abundance of ARGs and the abundance of microorganisms, the microorganisms can be considered as potential hosts of ARGs [38]. As shown in Figure 4E and Table S9, the co‐occurrence network included 74 nodes (28 ARG subtypes and 46 ARG carriers) and 287 edges. In the present study, 46 bacteria affiliated with four phyla were identified as possible hosts of 28 ARGs belonging to eight ARG types. Among the 46 bacterial species, both *Bradyrhizobium* and *Solibacter* were potential hosts of 12 ARGs. Some bacterial species (e.g., *Phocaeicola vulgatus* and *Blautia obeum*) were positively correlated with nine ARG subtypes. Among the 28 ARGs, 18 ARG subtypes were associated with more than one potential host, for example, *atetO*, *tetQ*, and *tetW* were associated with 34 potential ARG hosts. *Firmicutes* was the most common phylum, accounting for 58.2% of the selected phyla. It was positively correlated with various ARG subtypes, especially tetracycline resistance gene (*tet32*), multidrug resistance gene (*MexF*), and MLS resistance gene (*lnuC*), and established close relationships with various *Firmicutes*. In addition, Bacteroidetes (*Bacteroides stercoris*, *Phocaeicola dorei*, *Phocaeicola vulgatus*, and *Pseudomonas putida*) were found to be negatively correlated with various resistance genes.

**Figure 5:**
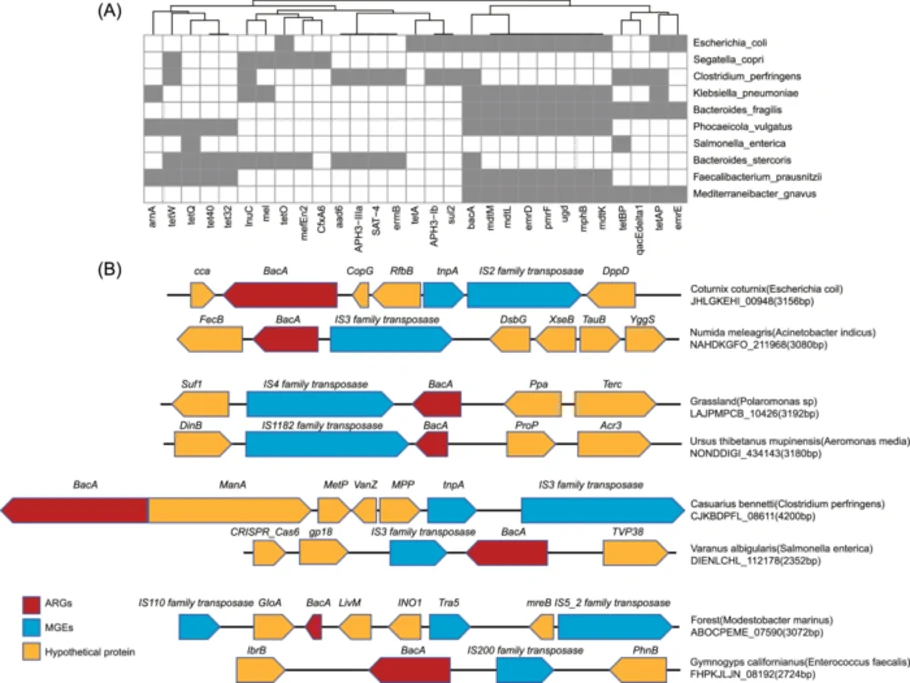
Distribution of high‐risk ARGs in transmission chains (vertebrates‐soil). (A) Binary heatmap of resistance gene resistance (gray, resistance observed; white, no resistance) assessed for the 10 most abundant metagenome assembled genomes (MAGs) recovered from metagenomic data. (B) Comparison of ARGs containing highly similar DNA fragments obtained from the macrogenomes of different host populations. MGEs, Mobile genetic elements.

We further investigated the exchange potential of ARGs between vertebrate gut microbiomes and the soil in their environment with metagenomic sequencing analysis. The genome of *E. coli* harbors a number of different ARGs, such as *bacA*, *mdtM*, *MarR*, *emrD*, and *pmrF* (Figure 5A). Thus, genomic analysis of *E. coli* suggests their potential for multidrug resistance in the vertebrate gut microbiome and the sympatric soil microbiome. Then, we evaluated the potential for horizontal gene transfer (HGT) in the flanking gene sequences of contigs containing *bacA*. Mobile genetic elements (MGEs) responsible for HGT potential were detected in multiple hosts of resistant bacteria containing bacA flanking groups, of which the most detected were transposases, mainly composed of IS and Tn families. Notably, we identified eight contigs that contained both *bacA* and MGEs, with five of these having *bacA* directly adjacent to an MGE, indicating potential mobility. Additionally, we found two instances of *bacA* transfer between mammals, avian species, and their sympatric soil microbial environments (Figure 5B). These results suggest that resistance genes and bacteria containing resistance genes may be transferred between soil biota and vertebrates.

## 3 DISCUSSION

Here, we collected 6508 fecal samples from 113 vertebrate species spanning seven classes with different feeding behaviors and habitats, by using 16S rRNA sequencing to investigate the ecological and biological drivers of gut microbial diversity and composition. We firstly evaluated the relative contribution of these drivers to the diversity of gut bacterial communities in vertebrate guts, and demonstrated that diet and climate factors have the strongest impact on gut microbial diversity. We also confirmed the effects of diet and climate on gut microbial composition and function. We then identified common antibiotic resistance and potential horizontal transfer of ARGs between terrestrial vertebrate gut microbiomes and their sympatric soil biological environmental samples using metagenomic datasets analysis. In conclusion, our study indicates both diet and climate factors play a critical role in driving the diversity and composition of gut microbiomes in vertebrates and reveals the potential threat that soil ARGs represent for animal health.

The abundant and diverse gut microbial communities live in the guts of both humans and animals are essential for the host physiology, ecology, and evolution [39, 40, 41, 42]. Gut microbiota are densely populated microbial communities that include many different types of bacteria that play essential and critical roles in regulation and adaptation of vertebrates to diverse lifestyles [43]. Here we firstly evaluated a range of host and environmental factors that may influence the gut microbial diversity in vertebrates. Among all factors considered, diet patterns were the strongest predictor of microbial community diversity, followed by climate factors.

Accumulating evidence has been pointing toward long‐term dietary patterns having profound effects on the diversity and structure of the trillions of microorganisms residing in animal guts [44]. As a specific example, two co‐evolution studies of mammals and their gut microbiota have found that both gut microbiota composition and functions are adapted to the animal diet (herbivorous, carnivorous, and omnivorous) [12, 45]. In another more recent study, the convergently evolved composition of the gut microbiomes in rodent species, despite their phylogenetic diversity, strongly suggests that diet is the major force shaping microbiota [46]. Here we observed that gut microbial diversity increased from carnivores to herbivores, consistently with previous studies [12, 47].

In the study at the phylum level, the content of Bacteroidetes was the highest in herbivores, and the content in carnivores was less than that in omnivores. Studies have shown that the content of Bacteroides phylum is related to phytophagy. Members of the Bacteroides phylum can digest cellulose and hemicellulose very well, and supply nutrients from them for the host. The Bacteroides phylum encodes more carbohydrate‐related enzyme families than other bacteria phyla [48, 49], which can digest cellulose and absorb related nutrients more efficiently [50]. In addition, we also noticed that Verrucomicrobia is prevalent in the intestines of many herbivores. The *Verrucobiotices* genus of the Verrucomicrobia phylum has been identified as a core taxonomic unit of the fecal microbiota of herbivores, indicating that it may play a key role in fiber digestion [51, 52]. The dominant bacterial genera found in herbivores include *Escherichia‐Shigella*, *Streptococcus*, *Cloacibacillus*, *Alistipes*, *Allobaculum*, and *Clostridium_XIVb*. In previous studies, most of these genera have been observed in microbial communities colonizing cellulose biomass [53, 54]. *Streptococcus* is one of the main starch‐degrading bacteria in animal intestines, capable of using cellobiose, pectin, xylose, and glucose as growth substrates [55]. Thus, the presence of this genus is explained. Additionally, similar studies on herbivores such as camels, sheep, goats, and cattle have also linked this genus to cellulose‐degrading bacteria [53, 56]. Moreover, the genus *Escherichia‐Shigella* is positively correlated with high‐fiber diets [57]. *Clostridium_XIVb* belongs to the *Lachnospiraceae*, which are bacteria specialized in decomposing complex plant materials (i.e., cellulose and recalcitrant substances in vegetables) [58]. In contrast, the main bacterial genera found in carnivores are *Acinetobacter*, *Pseudomonas*, *Aeromonas*, and *Fusobacterium*. *Fusobacterium* is typically associated with high‐fat and high‐protein diets and has been observed in various carnivores, previously classified as a carnivore‐specific bacterium [59]. This genus can utilize carbohydrates or amino acids to produce short‐chain fatty acids [60], and it has been demonstrated that *Fusobacterium* is usually present at higher concentrations in healthy carnivorous hosts [12, 61], which is consistent with our study.

The functions of intestinal microorganisms in carnivores are mainly in cell signaling and metabolic regulation. For example, ABC transporters, phosphotransferase systems, two‐component regulatory systems, and glutathione metabolism. The diet of carnivores is usually rich in high‐fat and high‐protein products, and carnivores can absorb protein and fat only through their own enzymes [62, 63], so the fatty acid degradation and ABC transporter pathways in the intestinal flora may play an important role in providing nutrition through the digestion and absorption of meat. Glutathione metabolism helps maintain normal immune system function, has antioxidant properties, and integrates detoxification processes [64], which is essential for carnivores. In addition, the phosphotransferase system (PTS)‐bacterial phosphoenolpyruvate (PEP) is a complex device present in bacteria that catalyzes the transport and phosphorylation of many monosaccharides, disaccharides, amino sugars, polyols, and other sugar derivatives [65]. Through this device, the intestinal microbiota may affect the energy metabolism of carnivores through the transport and phosphorylation of sugars produced by the intestinal microbiota. The functions of intestinal microorganisms in herbivores are mainly in protein synthesis. For example, (such as ribosomes, aminoacyl‐IRNA biosynthesis, tyrosine, and tryptophan biosynthesis). Ribosomes and aminoacyl‐tRNA biosynthesis are key processes that are indispensable for protein synthesis in cells. As an important machine in the cell, ribosomes are responsible for translating codons on mRNA into amino acid sequences to synthesize proteins [66]. Aminoacyl‐tRNA biosynthesis is the starting step of protein synthesis, combining specific amino acids with corresponding tRNA molecules to provide necessary carriers for subsequent translation processes [67]. At the same time, the biosynthesis of tyrosine and tryptophan is also one of the prerequisites for protein synthesis. These functions may help herbivorous intestinal microorganisms synthesize the required proteins and enzymes when processing plant foods, thereby improving food utilization efficiency. In addition, they increase the utilization of nitrogen sources by synthesizing aminoacyl‐tRNA, providing additional amino acid sources for the host. The ability to synthesize tyrosine and tryptophan helps microorganisms and host animals supplement these important physiologically active molecules in plant foods, supporting the host's immune system and overall health. These adaptive functions enable the gut microbiota of herbivores to effectively interact with their specific plant‐based diets and maintain the physiological balance and health status of the host.

Gut microbiota plasticity in response to environmental cues may allow hosts to rapidly adapt to ecological change [68, 69]. Recent evidence has suggested that gut microbiota is affected by both warming environment and changes to host ecology driven by climate variation [24, 70, 71]. We found the diversity and structure of gut microbiota among vertebrates are associated with climatic factors. Specifically, we noted the taxa of phylum Bacteroides and Proteobacteria are significantly correlated with climate factors. A previous study showed that the phylum Bacteroides and Proteobacteria, both gram‐negative bacteria, are strongly affected by temperature changes [72]. Interestingly, the phylum Proteobacteria could adapt to tolerate dryness and low temperatures by forming durable spores to protect itself under extreme conditions [73]. For instance, the abundance of Proteobacteria in freshwater lakes was enriched in winter, probably due to the strong adaptability of it to low temperature extremes [74]. In contrast, the phylum Bacteroides can adapt to relatively high temperature environments. For example, the abundance of Bacteroidetes in grasslands showed an increased trend with a rise in temperature [75]. Secondly, changes in the relative abundance of gut microbial taxa are often influenced by nutritional variations in food caused by different climatic regions. Some studies have found that the impact of different climatic regions on vertebrate gut microbiota is mainly attributed to differences in food resources [76]. Studies have shown that tropical diets are rich in fresh fruits and vegetables, rich in fiber and carbohydrates [77], which may be associated with increased levels of Bacteroidetes. Bacteroides are well‐known for their role in breaking down noncellulosic polysaccharides and pectins [78, 79], and they typically increase in high‐fiber or fruit‐rich diets [80, 81]. Members of the order Bacteroidales (especially the genus *Bacteroides*) have the largest repository of carbohydrate‐degrading activities, capable of fermenting various plant polysaccharides [82, 83]. The increase in these cellulose‐degrading/fiber‐degrading groups allows vertebrates living in tropical regions to optimally extract nutrients from their diet.

Co‐occurrence networks are a powerful tool in microbial research to understand the structure and function of microbial communities [84]. The co‐occurrence networks of different climate types in this study have different topological properties and complex species interactions. Studies have shown that the increase in the interaction of bacterial communities increases the size and complexity of the network, which reflects the fundamental differences between microorganisms in different samples [85]. The number of nodes, the number of links, and the average degree in the co‐occurrence network of vertebrate gut microorganisms show a tropical > subtropical > temperate > polar climate pattern, indicating that the stability of vertebrate gut microorganisms in tropical climates is higher than that in other climates [86], which may be related to the relatively low environmental pressure in the activity areas of vertebrates in tropical environments. Tropical climates tend to have fewer seasonal changes and extreme weather events, as well as abundant dietary resources [87]. Therefore, this study recommends strengthening the study of vertebrate gut microorganisms in polar regions. Polar ecosystems are particularly sensitive to climate change. Understanding the microbial composition of polar animals and their interactions with the environment will help reveal the response mechanism of biodiversity in the region. In addition, research in polar regions may also provide new perspectives and strategies for preventing and responding to the impacts of global climate change.

The development and spread of antibiotic resistance in bacteria is a growing global health threat to humans and animals [88, 89]. Soil is one of earth's largest reservoirs of ARGs (i.e., the soil antibiotic resistome), and is the habitat of many pathogens associated with clinical infections and animal disease outbreaks [90]. One of the most serious health concerns is the transfer of ARGs from soil to anthropogenic, animal, and plant settings, which would pose a severe threat to human and animal health [91]. However, there are still major unknowns associated with the transfer of ARGs and ARG‐containing bacteria between soil biological environments and vertebrates. In this study, we detected a large number of common antibiotic resistance genes between vertebrates and sympatric environmental samples, and in addition, we detected many high‐risk resistance genes (level I and level II) in them. Previous studies have shown that resistance genes associated with MGEs can frequently switch hosts within microbial communities, posing a high risk to the environment if the resistance genes are located on MGEs [92]. These genetic evidence suggest that these mobile resistance genes are transferred between pathogens and can be transferred between different species, potentially posing a risk to environmental health. Specifically, we explored the potential hosts of specific ARGs by annotating contigs carrying *bacA* and found that there may be horizontal transfer of *bacA* genes between the gut microbiome of *Ursus thibetanus mupinensis* and sympatric grassland soil biological environmental samples, and between the gut microbiome of *Gymnogyps californianus* and sympatric forest soil biological environmental samples. The *bacA* gene is associated with bacitracin resistance, suggesting a widespread use of this antibiotic. It has already been reported that the bacitracin resistance gene can be transferred to humans through close contact with ruminants [93]. The use of bacitracin as a growth promoter in the veterinary practice has significantly contributed to animal health, welfare, and performance, as well as to the overall productivity of the industry. However, bacitracin is banned as a feed additive in livestock farming by most countries. Based on our findings, we recommend that the relevant government authorities increase the surveillance on how this drug is being procured. Our findings highlight the health threats of soil bioenvironments harboring ARGs. We identified forested areas where the control of soil antibiotic resistance needs to be prioritized.

Our study must highlight some potential limitations. Given the extreme diversity and spatial variability of soil microbiomes [94], the approach employed in our study may oversimplify the complexity of soil microbial communities. The variability in soil microbiome composition even at small spatial scales (e.g., a few centimeters) suggests that collecting soil and gut microbiomes from a broad “same area” may not accurately represent the specific soil microbes to which vertebrates are exposed Group. This may mean that the observed overlap in antibiotic resistance genes between soil and gut microbiomes may not necessarily indicate true horizontal gene transfer events, but may reflect the widespread and abundant presence of these ARGs in natural environments. Horizontal gene transfer refers to the transfer of genes between microorganisms (rather than between generations). If true horizontal gene transfer events occur, then we would expect to find the same antibiotic resistance genes in microbiomes in different environments. However, if the spatial scale of the samples is too large, it may be found that the overlap of ARGs reflects the widespread occurrence of these genes in the natural environment. If ARGs are widespread in the environment, especially in soil, then this gene overlap may be more due to the prevalence of these genes in different microbial communities rather than actual horizontal gene transfer. Such prevalence may reflect the adaptability of these genes under natural selection and environmental pressures.

Furthermore, we propose some potential future research directions to further our understanding of the observed phenomena. First, in addition to dietary and climatic factors, research on the effects of animal gut microbiomes has increasingly focused on broader environmental characteristics. Studies using human and urban microbiomes as examples showed that air pollution (e.g., PM10) and environmental metabolites/chemicals could also significantly affect the composition and function of the gut microbiome [95, 96]. These environmental factors may affect the microbiome through multiple pathways, such as how tiny particles in air pollution enter the body through the respiratory tract, thereby affecting the stability and diversity of the gut microbiome. Or chemicals presented in the environment may enter directly into the food chain, ultimately affecting the gut microbiome of humans and other animals. These factors may vary across geographic locations and populations, so the impact of these environmental factors needs to be taken into account when studying the gut microbiome. In future studies, taking into account broader environmental characteristics will be critical to fully understand the formation and function of the animal gut microbiome. Second, it is recommended to conduct long‐term longitudinal studies to observe changes in gut microbial diversity over time in different vertebrate populations. Through this long‐term tracking, we can better understand the evolutionary trends of gut microbes and reveal their relationship with individual animal growth, health, and environmental factors. Finally, it is recommended to conduct experimental verification to determine the association between observed environmental factors and animal gut microbes. For example, animal model experiments can be designed to observe the effects of specific environmental factors (such as dietary habits and habitat climate) on intestinal microbes. These experiments help verify the causal relationship between environmental factors and microbial changes and gain a deeper understanding of the mechanisms of how they affect host health. These future research directions will help enhance our understanding of the interaction between animal intestinal microbes and the environment, and provide a more effective scientific basis for animal health management and environmental protection.

## 4 CONCLUSION

In conclusion, this is one of the largest studies of gut microbiota in vertebrates in terms of the number of individual samples collected globally. Our results can help to identify the major modulators of the diversity and structure of gut microbiomes. Our findings confirm that diet patterns and climate factors play key roles in promoting specific taxa in vertebrate gut microbiota. In addition, we comprehensively deciphered the common antibiotic resistomes of vertebrates and their sympatric soil biological environment samples, and found evidence of potential horizontal transfers of the *bacA* gene. These results significantly advance our knowledge of the diversity and structure of gut microbiomes in vertebrates and their association with environmental factors, and provide crucial insights to better manage the soil ARG pool.

## 5 MATERIALS AND METHODS

### 5.1 Study cohorts and data retrieval

We systematically searched existing literature (Google Scholar, Web of Science) for the following keywords: [“16S”] AND [“microbiome” or “microbiota”] AND [“V4” or “V3‐V4”] OR [“16S”] AND [“microbiome” or “microbiota”] AND [“V4” or “V3‐V4”] AND [“gut” or “fecal” or “intestines” or “cloaca”] OR [“16S”] and [“microbiome” or “microbiota”] AND [“V4” or “V3‐V4”] AND [“gut” or “fecal” or “intestines” or “cloaca”] AND [“vertebrate”] OR [“16S”] AND [“microbiome” or “microbiota”] AND [“V4” or “V3‐V4”] AND [“gut” or “fecal” or “intestines” or “cloaca”] AND [“mammal“ and “bird/avian” and “fish” and “amphibian” and “reptile” etc.]. We identified additional articles [97] through references cited in the retrieved results. We obtained 12,640 samples based on 16S rRNA gene sequencing from 99 published articles up to September 2022. We then retained samples with known hosts and confirmed origins for further analysis.

We then performed quality control for experimental animal, sample type, amplification primer and sequencing method as follows: (1) Samples from all vertebrates except livestock and poultry; (2) Samples of distal gut microbiota or fresh feces were retained; (3) Collected samples were not treated (such as antibiotic therapy) in a way that affected the composition of gut microbiome; (4) Samples were amplified using the V3‐V4 or V4 hypervariable regions of the 16S rRNA gene; (5) Samples were sequenced with amplicon sequencing on Illumina platform; (6) Number of samples at each location was greater than 8. This resulted in a data set of 6508 samples spread across seven continents (Figure S1). Species name, species category (class and order), diet information (carnivorous, herbivorous, and omnivorous), habitat type (terrestrial, aquatic, and amphibia), captivity status (wild or captive), threatened status (as indicated by the IUCN Red List for July 2022, least concerned: LC; near threatened: NT; vulnerable: VU; endangered: EN; critically endangered: CR; Extinct in the Wild: EW), sampling location, sampling climate zone, average earth surface temperature at the sampling point, and sampling year are included in Table S1. In addition, we further searched and collected the body size and gut length of 113 species using Google Scholar, PubMed, and Wikipedia, and included them in Table S2.

For the collection of metagenomes, we first collected a large number of soil metagenomes from around the world based on the work of Ji M et al. [98], Bahram et al. [99], and Delgado‐Baquerizo et al. [100]. Based on the region where soil metagenomic samples were collected, we can accurately search for terrestrial vertebrate intestinal metagenome samples in the same region on Google Scholar and Web of Science websites. Finally, we obtained a total of 487 vertebrate and 203 soil environmental metagenomic sequencing samples that were published up until December 2022 (Figure S1). Sampling information is included in Table S6.

### 5.2 16S rRNA sequencing analysis

We processed sequence data separately for each study to avoid potential batch effects through analyzing all the studies together according to Ho et al [101] and Zhou et al [102]. Paired‐end sequencing reads and quality control were merged using VSEARCH software [103]. Barcodes and primer sequences were removed using Cutadapt 4.1 [104]. For different hypervariable region sequences, other hypervariable regions were removed and only the V4 region was retained [105]. Afterward, the processed amplicon sequences of different hypervariable regions were merged for subsequent analysis. We also provide detailed information on sequencing details of 6508 samples, including the insert size, numbers of sequences and bases before and after quality control (Table S10).

Assembled sequences were de‐replicated, sorted, and clustered into OTUs at 97% identity using VSEARCH following standard UPARSE pipeline parameters [106]. Chimeric sequences were removed using UCHIME (implemented in VSEARCH) [107] and reference‐based combination (using the nonredundant SILVA SSU Ref database ver.123 as a reference). Each 16S rRNA gene sequence was assigned a taxonomic identity with the Ribosomal Database Project (RDP) classifier and compared against the Silva 16S rRNA gene database (version SSU128) using a confidence threshold of 70% [108]. OTU‐table and taxonomy‐table files were created using custom scripts.

The aligned results in usearch table format were directly converted to Biological Observation Matrix (BIOM) format using BIOM 2.1.5 [109]. Metadata was added to BIOM‐Format 1.3.1 using biom convert for community diversity analysis using QIIME2 (Version 2021.02). Alpha diversity calculations were done using Shannon index (quantitative community richness) and Observed OTUs (qualitative community richness). Beta diversity analysis was performed to assess dissimilarities in microbial communities between groups using Jaccard distance (qualitative community dissimilarity) and Bray‐Curtis distance (quantitative community dissimilarity).

### 5.3 Metagenomic sequencing analysis

Before assembly, all metagenomic sequencing analysis reads were assessed with FastQC (v0.11.8) for quality control and quality trimmed using Trimmomatic (v0.39) with the parameters “ILLUMINACLIP:TruSeq. 2‐PE.fa:2:40:15 LEADING:3 TRAILING:3 SLIDINGWINDOW:4:20 MINLEN:50.” Host (human) DNA removal was done by alignment against host genomes using BWA‐MEM (v2.2.1) [110] and SAMtools (v1.6) [111]. Reference genomes of the hosts are Hg38 (GCF_000001405.26) for human. The high‐quality clean reads were then assembled into contigs using the MEGAHIT software (v1.2.9) with the default settings [112]. Genes were predicted from the assembled contigs using the Prokka software pipeline (v1.14.6) [113]. To construct a nonredundant gene catalog, predicted gene sequences were clustered into homologous groups using CD‐HIT (v4.8.1) [114] and parameter settings of: ‐aS 0.9 ‐c 0.95. Kraken2 (v2.1.3) was utilized for species classification with Standard 72GB database [115]. A correction was performed using Bracken [116], resulting in a species abundance table.

Antibiotic resistance genes were annotated with the Structured Antibiotic Resistance Genes (SARG) database using the ARGs‐OAP (v3.2.2) pipeline [117]. Abundance of each ARG subtype or type was normalized with cell copy number in each metagenomic data (as relative abundance), which is recommended as the universal unit in environmental research [118]. The risk rank of ARGs and the ARGs risk rank index were evaluated using arg_ranker (v3.0.1) against the SARG database with the following parameters: ‐i $INPUT ‐kkdb $kraken2_standard [119]. To evaluate the potential mechanisms of ARG movement, contigs carrying ARGs were compared to the ISfinder database [120] and the ACLAME database (v0.4) [121] to investigate the co‐occurrence structure of ARGs and MGEs in contigs. E‐value cutoffs were 1e − 5, identity ≥80%, and alignment length ≥80% [122].

### 5.4 Multiple regressions on matrices (MRMs)

We used MRMs to study host Phylogeny, habitat type (aquatic, amphibious, and terrestrial), diet (carnivorous, omnivorous, and herbivorous), threatened status (LC, NT, VU, EN, CR), captivity status (wild or captive), climate (annual average temperature), and geographical location (latitude and longitude). In the order listed above, the habitat type from 0 to 2, the diet was coded from 0 to 2, the threatened status from 5 to 1, the binary variables for captive status were set as 0 (wild) and 1 (captive). Gower distances among communities were separately calculated for all independent variables, while Euclidean distances were calculated for Shannon index differences, and Bray‐Curtis distances were calculated to estimate beta diversity among gut communities. For MRMs using the EcoDist R package [123], the effect size and significance were obtained by comparing actual data to a random arrangement (*n* = 1000 for all analyses).

### 5.5 Statistical analyses

We compared gut bacterial community characteristics with Kruskal–Wallis (multiple group comparison) or two‐tailed Wilcoxon rank‐sum (pairwise comparison) tests. A false discovery rate (FDR)‐corrected *p* < 0.05 was considered statistically significant for these tests. The R package “ggplot2” was used to visualize box plots of data distributions [124]. Variations in the bacterial community compositions and functions of different samples were evaluated by Nonmetric MultiDimensional Scaling (NMDS) based on the Bray–Curtis distance. Random forest regression was performed using the randomForest package in R to assess the relative importance of different factors on the gut microbial diversity of vertebrates [125].

We used a dated host phylogeny for all species from http://timetree.org. We conducted geographic analysis using the ArcGIS 10.2 software (Environmental Systems Research Institute Inc., Redlands, USA). The LEfSe analysis was performed with the following parameters: the alpha value for factorial Kruskal–Wallis test among classes was <0.05, and the threshold on the logarithmic Linear Discriminant Analysis (LDA) score for discriminative features was 4.0. Functional profiles were inferred with PICRUst (version 2.5.1). The inferred genes and their functions were aligned with the Kyoto Encyclopedia of Genes and Genomes. PICRUSt‐predicted functional profiles were analyzed by STAMP (version 2.1.3), with significance assessed by Welch's *t*‐test with Storey's FDR (reported *p < 0.05, effect size >0.5). UpSet plots were visualized using the Upset server (http://www.bioinformatics.com.cn/plot_basic_upsetR_plot_009).

In this study, we analyzed the co‐occurrence of OTUs in the gut microbiome of vertebrates in different climatic regions. To reduce network complexity and facilitate identification of core gut communities, we selected OTUs with a relative abundance >0.1% and present in more than 50% of the samples for analysis. For all networks, we performed Spearman rank correlation analysis on OTUs and explored the co‐occurrence patterns of gut microbial communities based on strong correlations (*ρ* > 0.6) and significant correlations (*p* < 0.05) [126]. Topological features of each network were also calculated. These features include modularity (M), average degree (avgK), average clustering coefficient (avgCC), and average path distance (GD). In addition, to investigate the co‐occurrence patterns between ARGs and identify potential hosts of ARGs, we constructed networks as previously described [127]. Species with mean relative abundance >0.1% and present in >50% of samples in a given group and the top 50 relative abundance ARG subtypes were used. A correlation matrix was generated by calculating all possible pairwise Spearman rank correlations between ARG subtypes and species. Correlations between two items were statistically robust if the Spearman correlation coefficient (*ρ*) > 0.6 and the *p* value < 0.05. All networks were visualized using the Fruchtermann‐Feingold layout of the interactive platform Gephi version 0.9.7 [128].

## AUTHOR CONTRIBUTIONS

**Lijing Bai** and **Kai Xing**: Conceptualization; methodology; investigation; writing‐review & editing; supervision. **Yong Xie** and **Songsong Xu**: Methodology; software; investigation; data curation; formal analysis; writing‐original draft. **Yufei Xi**, **Zixin Li**, **Erwei Zuo**, and **Kui Li**: Investigation; data curation. All authors have read the final manuscript and approved it for publication.

## CONFLICT OF INTEREST STATEMENT

The authors declare no conflict of interest.

## ETHICS STATEMENT

No animals or humans were involved in this study.

## Supporting information

The online version contains supplementary figures and tables available.

**Figure S1:** Locations where 16 s samples and metagenomic data were collected across the globe.
**Figure S2:** Phylum‐level composition of gut community diversity shown by host phylogeny and with other relevant host metadata.
**Figure S3:** Evaluation of the effects of different factors on the diversity of vertebrate gut microbial communities.
**Figure S4:** Random forests were used to determine the relative importance of different factors.
**Figure S5:** Diversity of host gut microbiota collected from different diets and years.
**Figure S6:** The diversity of gut microbiota in hosts with the same diet and phylogenetics in different climatic regions.
**Figure S7:** The relative abundance of gut microbiota in vertebrates at different levels.
**Figure S8:** A symbiotic network constructed by gut communities of vertebrates from different climatic regions.

**Table S1:** Host samples and corresponding information for gut bacterial community 16S rRNA gene sequencing samples recovered from global vertebrates.
**Table S2:** Corresponding information on body size and intestinal length of the 100 hosts collected.
**Table S3:** Statistical data on the Alpha diversity of vertebrate gut microbes collected in different years.
**Table S4:** Permutational multivariate analysis of variance (PERMANOVA).
**Table S5:** Topological properties of the co‐occurrence network of different climate regions at the OTU level.
**Table S6:** Corresponding information for metagenomically sequenced samples of vertebrate gut bacterial communities worldwide and soil metagenomically sequenced samples.
**Table S7:** Risk levels of 1426 ARGs identified using ARG‐ranker.
**Table S8:** The percentage of each risk level in each sample.
**Table S9:** Details of potential ARG hosts and the corresponding ARG subtypes they harbored.
**Table S10:** Detailed information on sequencing details of 6508 samples from 113 vertebrate species.

## Data Availability

The data that supports the findings of this study are available in the supplementary material of this article. All sample metadata used in this study is provided in Supplementary Table. All results in the manuscript can be reproduced using the metadata provided in Supplementary Table and the code provided on GitHub at https://github.com/xieyg34/Global-meta-analysis. Supplementary materials (figures, tables, graphical abstract, slides, videos, Chinese translated version, and update materials) may be found in the online DOI or iMeta Science http://www.imeta.science/imetaomics/.
