# Supplementary material for: Global meta‐analysis reveals the drivers of gut microbiome variation across vertebrates
Source: IMetaOmics. 2024 Oct 4;1(2):e35. doi: 10.1002/imo2.35 (PMC12806492; doi:10.1002/imo2.35)
Supplement: Supplementary file 1 — Figure S1: Locations where 16 s samples and metagenomic data were collected across the globe. Figure S2: Phylum‐level composition of gut community diversity shown by host phylogeny and with other relevant host metadata. Figure S3: Evaluation of the effects of different factors on the diversity of vertebrate gut microbial communities. Figure S4: Random forests were used to determine the relative importance of different factors. Figure S5: Diversity of host gut microbiota collected from different diets and years. Figure S6: The diversity of gut microbiota in hosts with the same diet and phylogenetics in different climatic regions. Figure S7: The relative abundance of gut microbiota in vertebrates at different levels. Figure S8: A symbiotic network constructed by gut communities of vertebrates from different climatic regions. [file IMO2-1-e35-s001.docx]

**Supplementary information to**

# Global meta-analysis reveals the drivers of gut microbiome variation across vertebrates

**Running title:** Environmental drivers of vertebrate gut microbiome variation

Yong Xie^1#^, Songsong Xu^2#^, Yufei Xi^3^, Zixin Li^1^, Erwei Zuo^1^, Kai Xing^2^*, Lijing Bai^1,4^*, Kui Li^1^

^1^ Shenzhen Branch, Guangdong Laboratory of Lingnan Modern Agriculture, Agricultural Genomics Institute at Shenzhen, Chinese Academy of Agricultural Sciences, Shenzhen 518124, China

^2^ College of Animal Science and Technology, China Agricultural University, Beijing 100193, China

^3^Animal Science and Technology College, Beijing University of Agriculture, Beijing 102206, China

^4^ Animal Breeding and Genomics, Wageningen University & Research, Wageningen 6708 PB, The Netherlands.

^#^These authors contributed equally:Yong Xie, Songsong Xu

^*^Corresponding authors: xk@cau.edu.cn (Kai Xing), [bailijing@caas.cn](mailto:bailijing@caas.cn) (Lijing Bai)


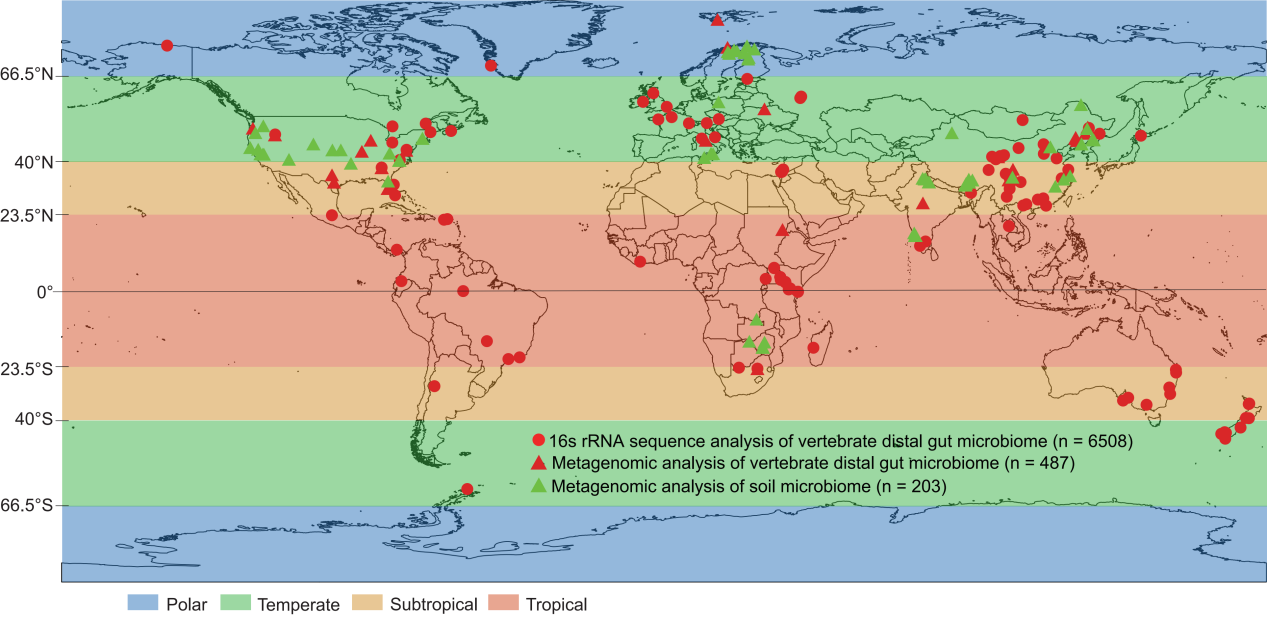


**Figure S1 Locations where 16s samples and metagenomic data were collected across the globe.**


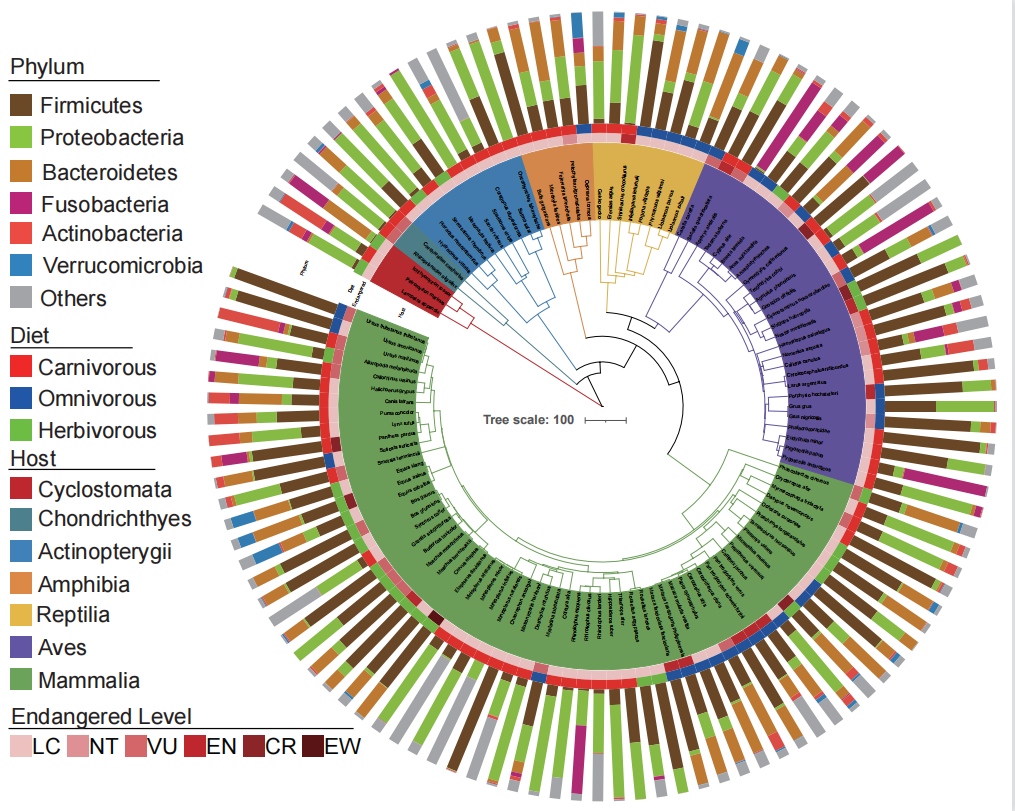


**Figure S2 Phylum-level composition of gut community diversity shown by host phylogeny and with other relevant host metadata.** The time-calibrated host phylogenetic tree was obtained from http://timetree.org and the colors of the branches represent the host class (red = *Cyclostomata*; dark blue = *Chondrichthyes*; light blue = *Actinopterygii*; dark yellow = *Amphibia*; light yellow = *Reptilia*; purple = *Aves*; green = *Mammalia*). The data mapped onto the tree (from the inner to outer circles) show the threatened status (obtained from the IUCN Red List in July 2022), host diet, and the relative abundances of bacterial phyla in each host. Relative abundances are averages estimated by subsampling OTUs from all samples of each host species (subsampling to 10,000 for each host species).

**
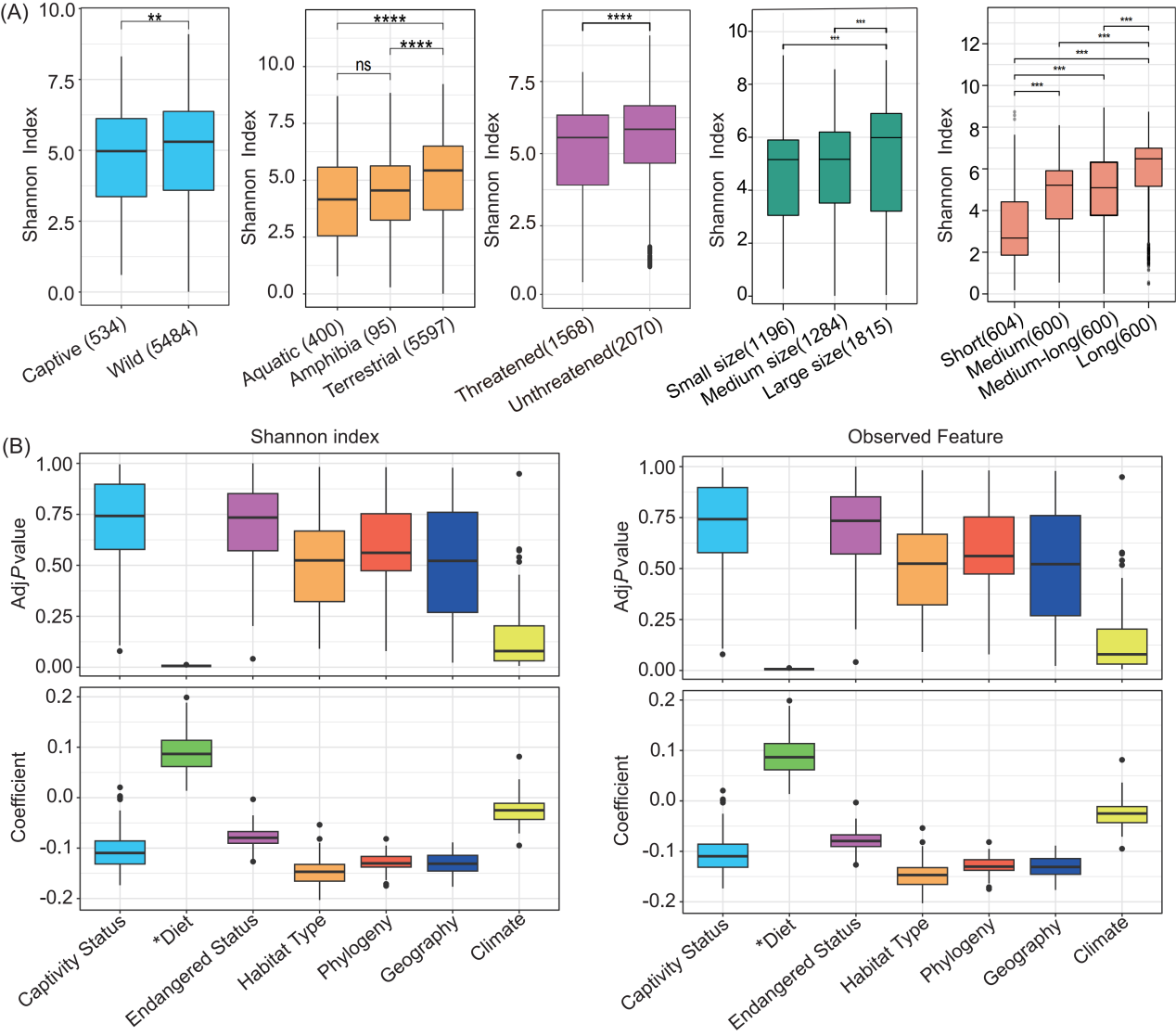
**

**Figure S3 Evaluation of the effects of different factors on the diversity of vertebrate gut microbial communities.** (A) The alpha diversity level of the gut community was grouped by host vertebrate captive status, habitat type, threat status, body size, and gut length. FDR-corrected Wilcoxon rank sum tests were used to determine significance. ***: *p* < 0.001, **: *p* < 0.01, *: *p* < 0.05. (B) The plots show the BH-adjusted *p* values (Adj. *p* value) and partial regression coefficients (Coef.) for multiple regression on matrices (MRMs) tests used to determine how much microbial diversity variance was explained by host diet, captivity status, geographic location, habitat, phylogeny, climate and threatened status. Asterisk denotes significance (Adj. *p* < 0.05 for ≥ 95% of dataset subsets). Box centerlines, edges, whiskers, and points signify the median, interquartile range (IQR), 1.5×IQR, and >1.5 IQR, respectively.

**
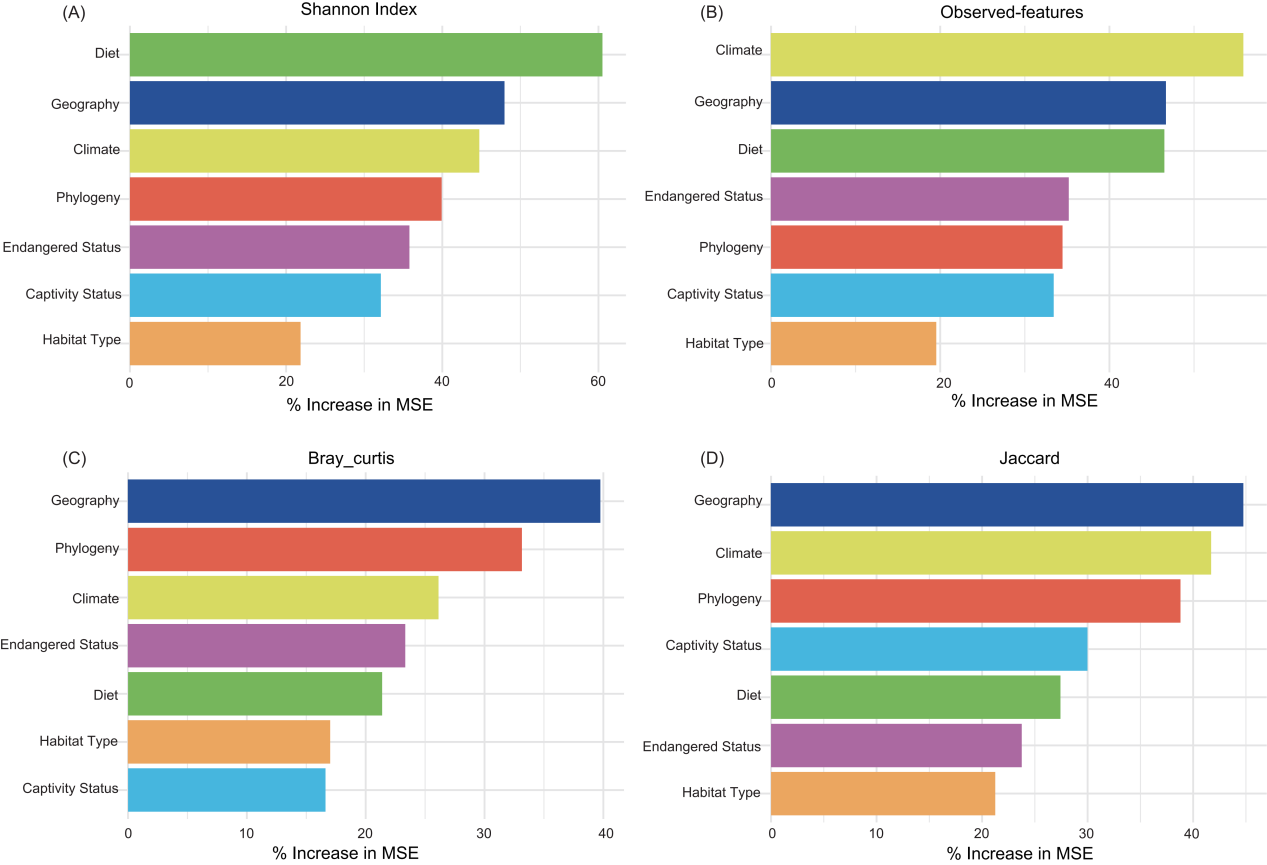
Figure S4 Random forests were used to determine the relative importance of different factors.** By randomly assigning values to each predictor variable, if the predictor variable is more important, the error of the model prediction will increase after its value is randomly replaced. "%IncMSE" stands for increase in mean squared error.


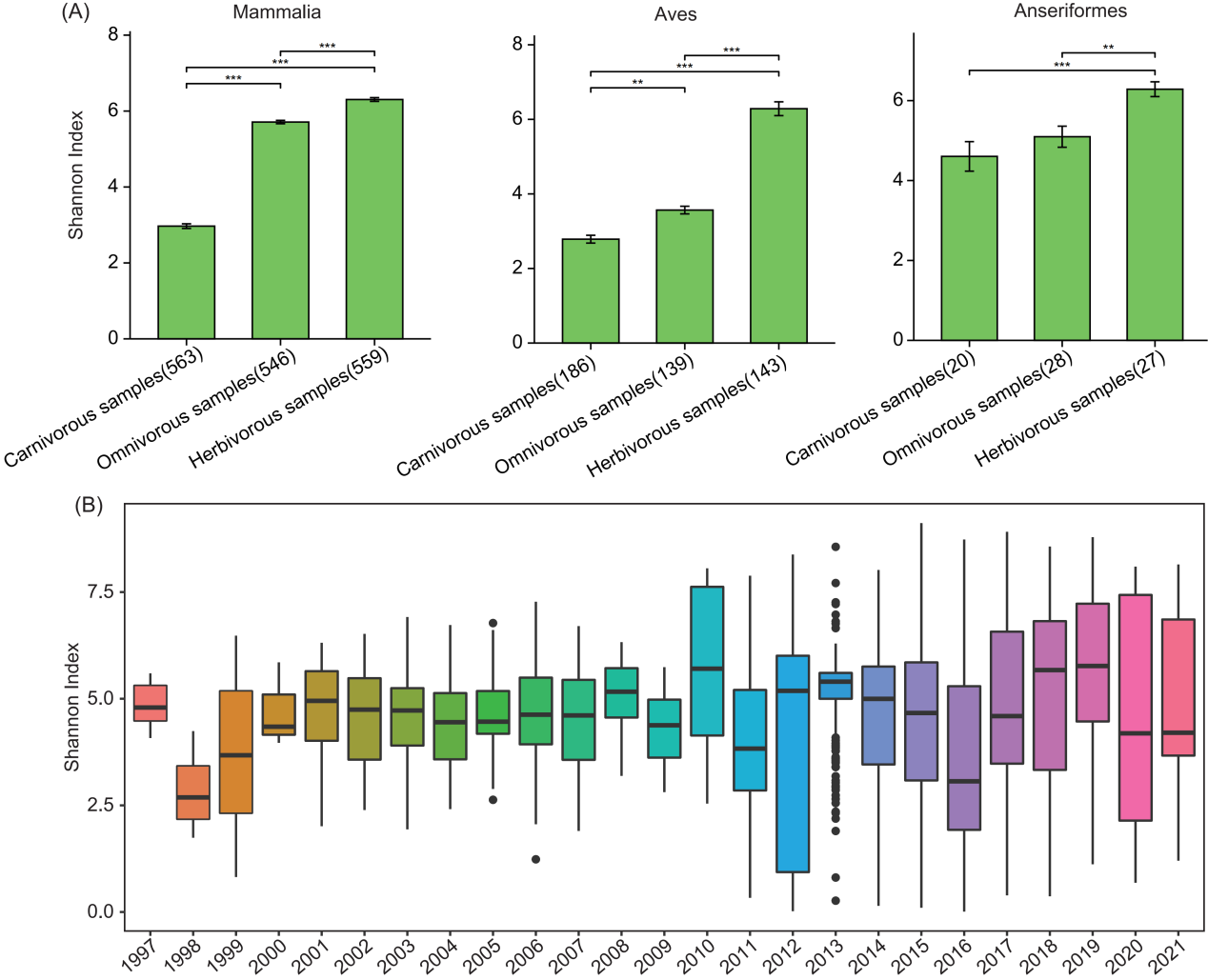


**Figure S5 Diversity of host gut microbiota collected from different diets and years.** (A) Diversity of gut microbiomes in vertebrates with different diets (same class or order). (B) Microbial diversity of vertebrate gut microbial samples collected in different years is shown.


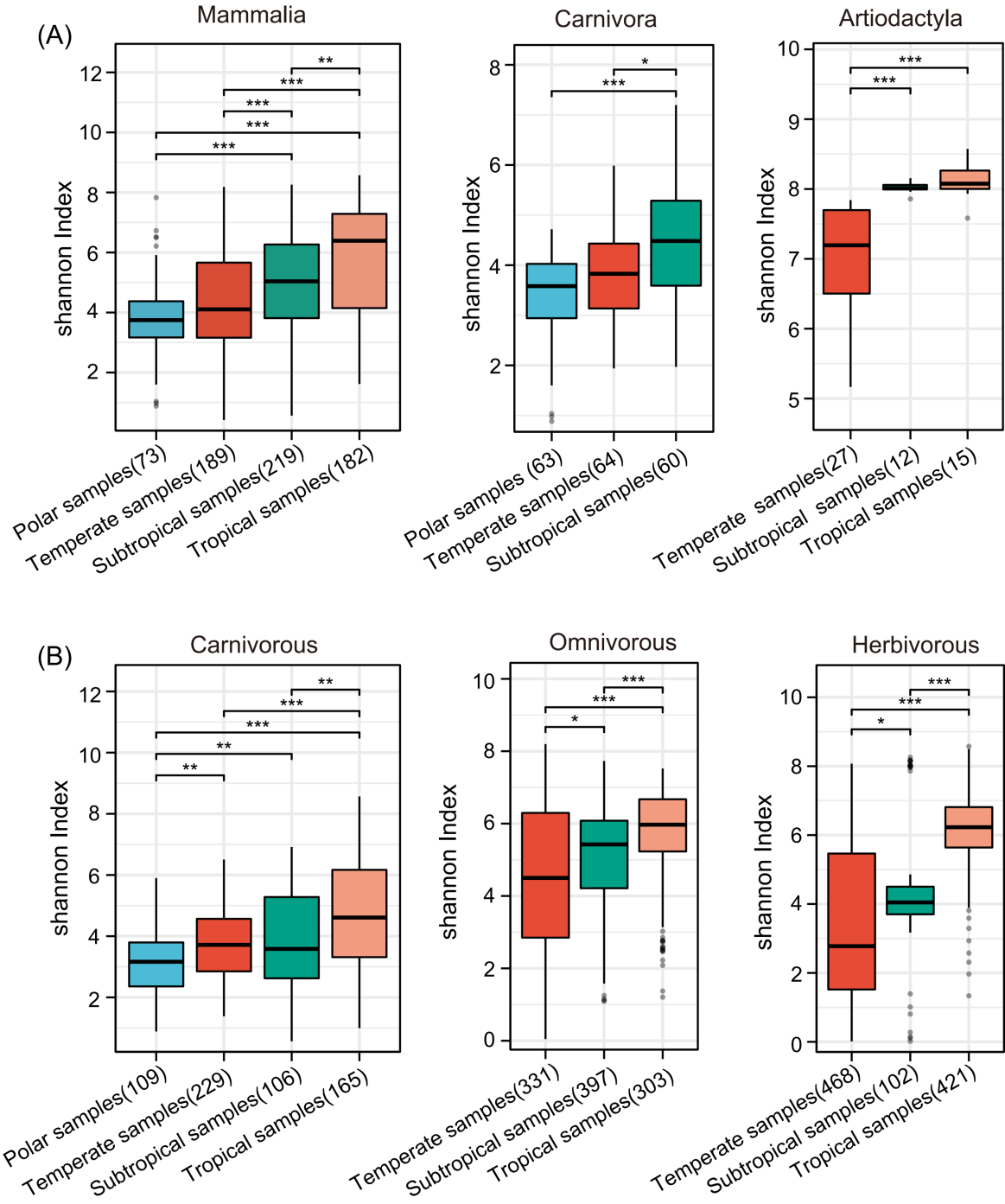


**Figure S6 The diversity of gut microbiota in hosts with the same diet and phylogenetics in different climatic regions.** (A) Diversity of gut microbiomes in vertebrates from different climate regions (same order or class). (B) Diversity of gut microbiomes in vertebrates from different climate regions (same diet habits).


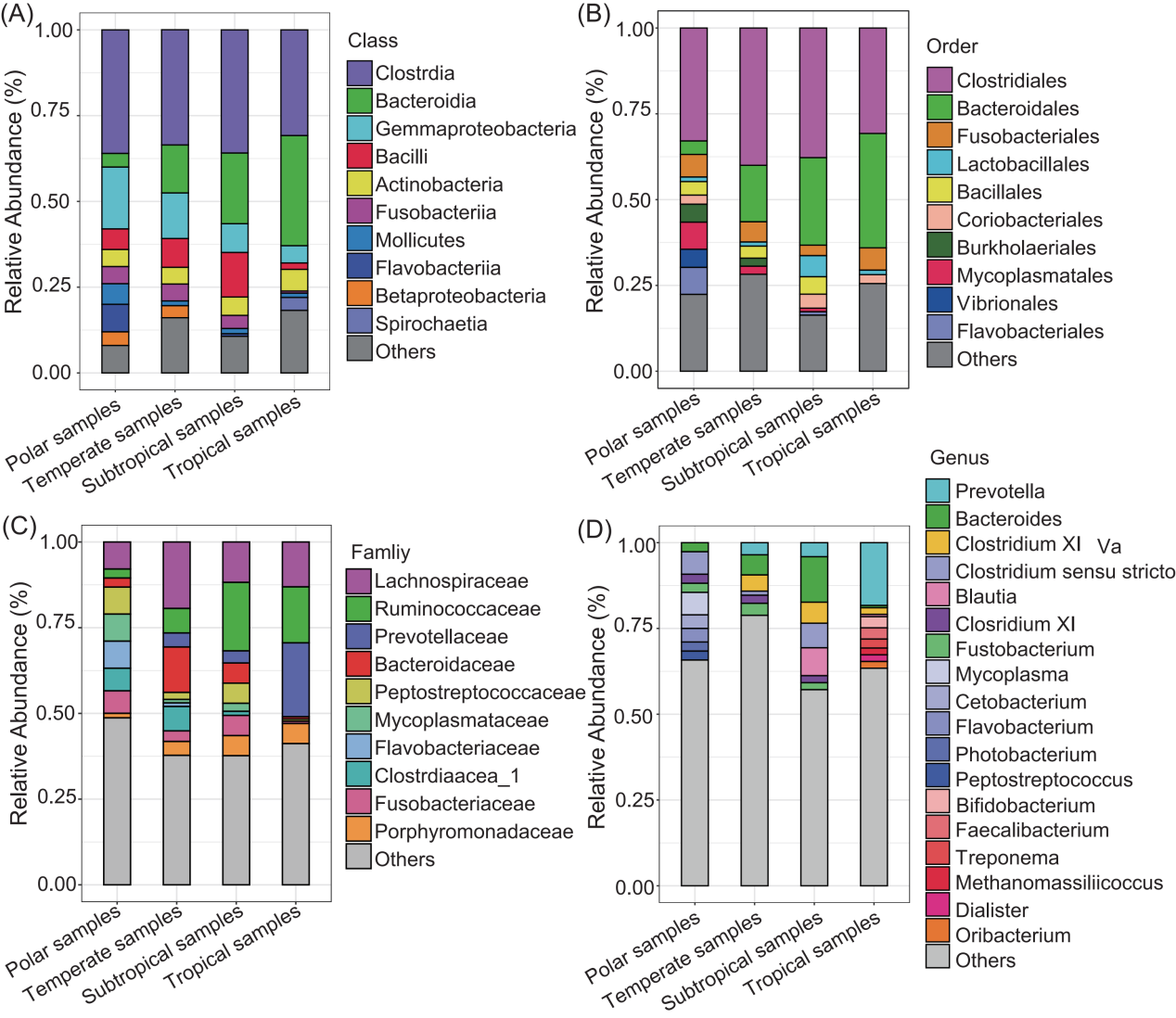


**Figure S7 The relative abundance of gut microbiota in vertebrates at different levels.** (A-D) Relative abundance of intestinal flora analyzed at the level of class (A), order (B), family (C), and genus (D).

**
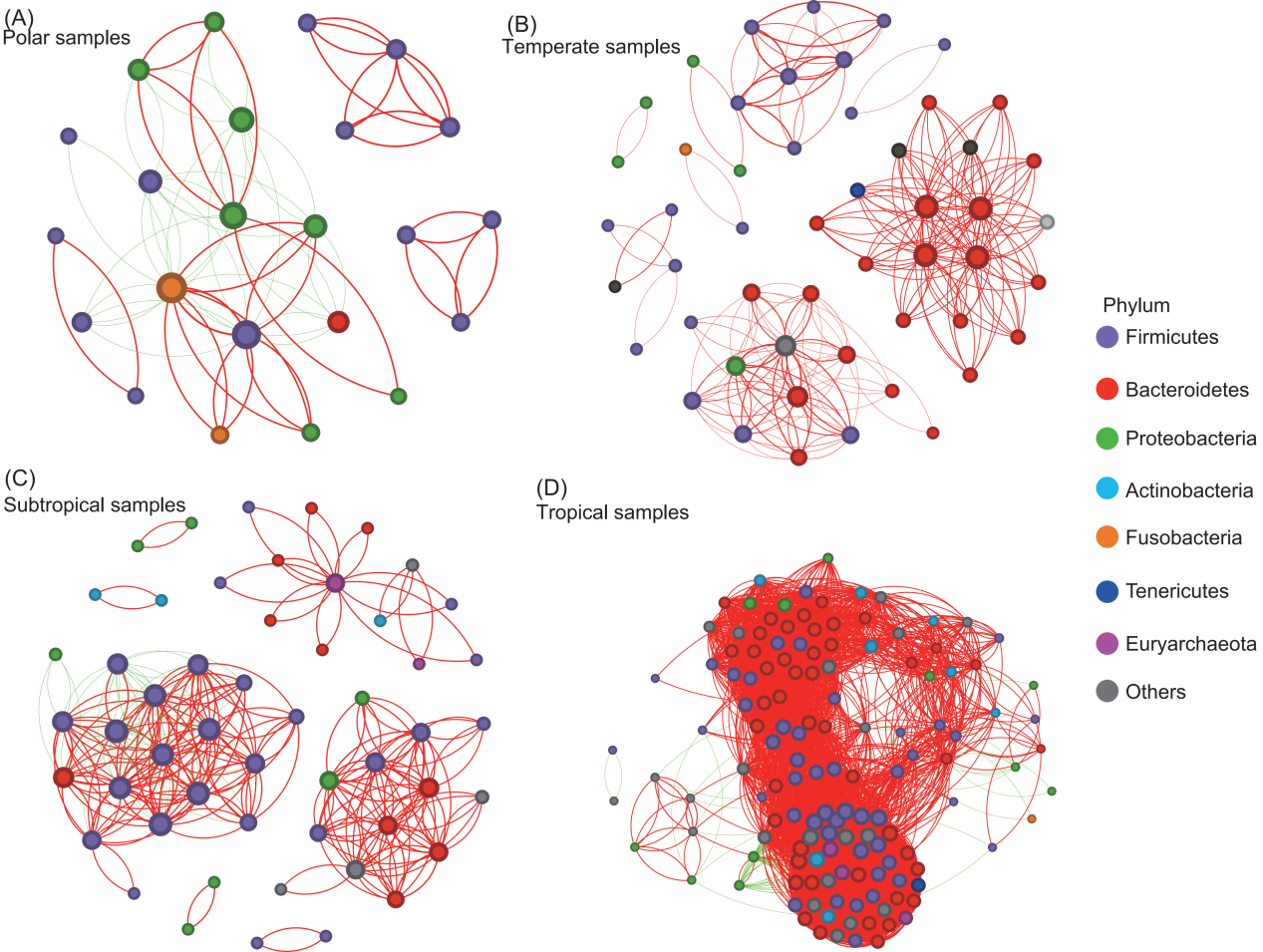
Figure S8 A symbiotic network constructed by gut communities of vertebrates from different climatic regions.** (A-D) Co-occurrence network constructed by correlating vertebrate intestinal communities in polar (A), temperate (B), subtropical (C) and tropical regions (D). The size of each node is proportional to the number of connections. Nodes in the network are colored by gate. Positive interactions are represented by red and negative interactions are represented by green edges.
